# The role of *Artemisia turanica* extract on renal oxidative and biochemical markers in STZ-induced diabetes in rat

**Published:** 2020

**Authors:** Hassan Bagheri Yazdi, Mousa-Al-Reza Hadjzadeh, Vida Hojati, Abdolhossein Shiravi, Sara Hosseinian, Gholamhassan Vaezi

**Affiliations:** 1 *Department of Biology, Damghan Branch, Islamic Azad University, Damghan, Iran*; 2 *Department of Physiology, School of Medicine, Mashhad University of Medical Sciences, Mashhad, Iran*; 3 *Division of Neurocognitive Sciences, Psychiatry and Behavioral Sciences Research Center, Mashhad, Iran*; 4 *Neurogenic Inflammation Research Center, Mashhad University of Medical Sciences, Mashhad, Iran*

**Keywords:** Diabetes mellitus, Artemisia turanica, Metformin, Oxidative stress

## Abstract

**Objective:**

The aim of the current study was to investigate the protective effect of *Artemisia turanica* (AT) against diabetes- induced renal oxidative stress in rats.

**Materials and Methods:**

Fifty male Wistar rats were randomly divided into five groups: control, STZ-induced diabetic rats, diabetic rats+ metformin, diabetic rats + AT extract, diabetic rats+ metformin+ AT extract. In the present study, diabetes was induced by a single-dose (55 mg/kg, ip) injection of streptozotocin (STZ). Diabetic rats were daily treated with metformin (300 mg/kg), AT extract (70 mg/kg) and metformin+ AT extract for 4 consecutive weeks. Tissue activities of superoxide dismutase (SOD) and catalase and the levels of malondialdehyde (MDA) and total thiol content were measured in kidney tissue. Serum concentrations of glucose, creatinine, and urea, as well as, lipid profile were also measured.

**Results:**

STZ significantly increased the levels of glucose, triglyceride, urea and MDA compared to the control group. Total thiol content, as well as, catalase and SOD activities showed significant decreases in diabetic group when compared with the control animals. Serum glucose, triglyceride, cholesterol and renal MDA showed a significant decrease and renal total thiol and the activities of antioxidant enzymes showed significant increases in AT+STZ group compared with the diabetic group. In diabetic rats received AT+ metformin, serum LDL and HDL, renal MDA and SOD and catalase activities significantly improved compared with the diabetic rats.

**Conclusion:**

These findings suggested that AT extract has therapeutic effects on renal oxidative damage and lipid profile in diabetes, that possibly may be due to its antioxidant and hypolipidemic effects.

## Introduction

Diabetic nephropathy is an important complication of diabetes mellitus that causes a high rate of mortality and morbidity throughout the world (Reutens and Atkins, 2011 ▶). The occurrence of renal failure in diabetic patients is mainly due to hemodynamic dysfunctions and is characterized by structural and functional abnormalities (Cheng and Harris, 2014 ▶). In diabetic patients, there are a wide range of glomerular and tubulointerstitial disorders including thickening of glomerular basement membranes, podocyte loss, enlargement of tubular basement membranes, interstitial fibrosis and tubular atrophy (Pourghasem et al., 2015 ▶). It is well established that oxidative stress plays a critical role in the development of diabetic kidney failure. Several pathways such as glycolysis, xanthine oxidase, and advanced glycation generate free radicals, have been considered main contributors to the pathogenesis of diabetic nephropathy (Forbes et al., 2008 ▶). Furthermore, increasing data suggests that chronic hyperglycemia-induced mitochondrial production of reactive oxygen species (ROS) may be an initiator for these pathogenic pathways (Forbes et al., 2008 ▶). In diabetic patients, the uptake of glucose enhances in different renal cell populations including podocytes, mesangial cells, and proximal tubular epithelial cells. Therefore, in addition to tissues such as the retina and neuronal and glial cells in peripheral nerves, kidneys are susceptible to conditions created during diabetes. Chronic hyperglycemia also accelerates the formation of advanced glycation end products (AGEs). During the formation of these products, ROS are produced and a cycle of ROS/AGE formation is developed in diabetes. The majority of AGEs are ultimately cleared by the kidneys and thereby interact with different renal cell populations (Kashihara et al., 2010 ▶). In mammal’s cells, there are various antioxidant systems that are involved in response to excess ROS generation. Among antioxidant systems, superoxide dismutase (SOD) is the most important antioxidant enzyme which is responsible for detoxification of superoxide radicals to hydrogen peroxide. Hydrogen peroxide is in turn decomposed to water and oxygen by another antioxidant enzyme, catalase. To date, the blockers of renin-angiotensin system (RAS) are one of the most effective drugs for treatment of diabetic renal disease (Chawla et al., 2010 ▶). In recent years, the use of herbal medicines with potent antioxidant and anti-inflammatory properties, has developed to minimize hyperglycemia and other metabolic disorders associated with diabetes. Due to an increase in triglyceride-carrying lipoproteins, chylomicrons and very-low-density lipoprotein, hypertriglyceridemia is a predominant abnormality of lipid metabolism in diabetes (Biesenbach, 1989 ▶). 

*Artemisia turanica *(AT) commonly known as “Dermane Ghermez”, belongs to Asteraceae (Compositae) family and grows mainly in northeast of Iran (Mozaffarian, 1998 ▶). The essential oil of AT contains some effective components including camphor, 1, 8-cineol, chrysanthenone, davanone, cis-verbenyl, 5, 7-methoxyflavone and oxygen containing monoterpenoids (Khayyat and Karimi, 2005 ▶). Different species of *Role of the potassium channels in vasorelaxant effect of asafoetida essential oil* possess different biological activities including antioxidant, anti-inflammatory, antimalarial, anti-fungal, cytotoxic and apoptotic properties (Yun et al., 2016 ▶; Hosseinzadeh et al., 2018 ▶; Behravan et al., 2006 ▶; Taherkhani et al., 2013 ▶). The current study was undertaken to determine whether *Artemisia turanica *aqueous-ethanolic extract could improve kidney dysfunction and oxidative stress in STZ- induced diabetic rats.

## Materials and Methods

**Preparation of plant extract**

Aerial parts of the plant were collected from Khaf (Razavi Khorasan province, Iran) and identified by a botanist in the Herbarium of School of Pharmacy, Mashhad University of Medical Sciences, Mashhad, Iran (specimen number 12572). The plant was washed, shade-dried and powdered. Then, the powder was macerated using 70% ethanol with occasional shaking and stirring. At the end, the filtered mixture was kept at 45°C for solvent evaporation to yield a blackish-brown concentrate. The prepared extract was kept at 4°C prior to use. 

**Animals**

Fifty male Wistar rats (weighed 200±20 g) were obtained from the Animal House of the School of Medicine, Mashhad University of Medical Sciences, Mashhad, Iran. All animals were maintained at 22±2^o^C with 12 hr light/dark cycles. All experiments on animals were done according to National Laws regarding the use/care of laboratory animals.

**Experimental design**

Diabetes was induced by a single injection of streptozotocin (STZ, 55 mg/kg, i.p. freshly prepared in normal saline) (Sigma-Aldrich, USA) (Dashtban et al., 2016 ▶). Control animals received equal volume of normal saline. Serum glucose concentration was measured in blood taken from the tail vein using glucose meter (Clever check, TD-4230), 3 days after diabetes induction. Rats with serum glucose level above 300 mg/dl were considered diabetic (Park and Han., 2012 ▶). AT extract (70 mg/kg) (Bagheri Yazdi et al., 2019 ▶) and metformin (Met, Samisaz company, Mashhad, Iran) (300 mg/kg) (Bagheri Yazdi et al., 2019 ▶) were orally (by gavage) administrated to diabetic rats for 28 days. The animals were randomly divided into five groups (n=8) as follows:

Control group, STZ-induced diabetic group, diabetic rats+metformin (Met+STZ) group, diabetic rats+AT extract (AT+STZ) group, diabetic rats+metformin+AT extract (Met+AT+STZ) group.

At the end of the experiment, serum samples were collected from the orbital sinus. Blood samples were centrifuged at 2000 rpm for 10 min for separation of serum and kept at -20°C for serum glucose, creatinine, urea and lipid profile measurement. Then, all animals were humanely killed and the left kidneys were quickly removed and conserved at -80°C for oxidative stress assessment.

**Assessment of serum biochemical parameters**

Serum concentration of glucose, creatinine, urea, triglyceride (TG), total cholesterol (TC), high-density lipoprotein cholesterol (HDL-C) and low-density lipoprotein cholesterol (LDL-C) were measured by commercial kits (Pars Azmoon, Tehran, Iran) based on manufacturer’s instructions. 

**Assessment of oxidative stress markers**

**Total thiol content measurement**

Thiols are organic compounds which contain a sulphydryl (-SH) group. DTNB (5, 5-dithio-bis-(2-nitrobenzoic acid) is used as a reagent for measurement of thiol groups. This reagent reacts with the SH groups to produce a yellow colored complex which has a peak absorbance at 412 nm. Briefly, in a cuvette, kidney homogena, and Tris-EDTA buffer were mixed and the absorbance was read at 412 nm against Tris-EDTA buffer alone (A1). Then, DTNB reagent (10 mM in methanol) was added to the mixture and after 10 min, the sample absorbance was read again (A2). The absorbance of DTNB reagent was also read as a blank (B). Total thiol content (mM) was calculated from the following equation (Hosseinian et al., 2017 ▶):

 Total thiol concentration (mM) = (A2-A1-B) ×1.07/0.05×13.6

**Malondialdehyde (MDA) measurement**

Lipid peroxidation of kidney tissues was assessed by measuring MDA which reacts with thiobarbituric acid (TBA) as a thiobarbituric acid reactive substance to produce a red colored complex with a peak absorbance at 535 nm. Briefly, 2 ml of TCA (trichloroacetic acid/TBA)/HCl reagent was added to 1 ml of homogenate. Then, 15 g TCA, 0.375 g TBA and 2 ml HCl were mixed and 2 ml of this mixture was added to 1 ml of kidney homogenate. Then, the mixture was heated for 50 min in a boiling water bath. After cooling to room temperature, the mixture was centrifuged at 1000 rpm for 10 min. The absorbance (A) of the colored layer was read at 535 nm. MDA concentration was calculated from the following equation (Hosseinian et al., 2018):

C (M) = A/1.56×10^5^

**Assessment of antioxidant enzymes activities**

Superoxide dismutase (SOD) activity in kidney tissues was determined by the method of Madesh & Balasubramanian (Madesh and Balasubramanian, 1998 ▶). In a colorimetric assay, the SOD activity was measured at 570 nm. One unit of SOD was defined as the amount of enzyme required to inhibit the rate of MTT reduction by 50%. The results are shown as unit per milligram protein. Catalase activity was measured according to the method of Aebi with hydrogen peroxide (30 mM) as the substrate (Aebi, 1984 ▶). One unit of catalase activity was determined as the micromoles of the hydrogen peroxide consumed per milligram of protein sample.

**Statistical analysis**

The data is expressed as mean±SEM. One-way ANOVA followed by a *post hoc* Tukey comparison test was used to compare data. Statistical significance was considered as p<0.05. 

## Results

**The effect of AT extract and metformin on serum biochemical parameters**

**Figure 1:**
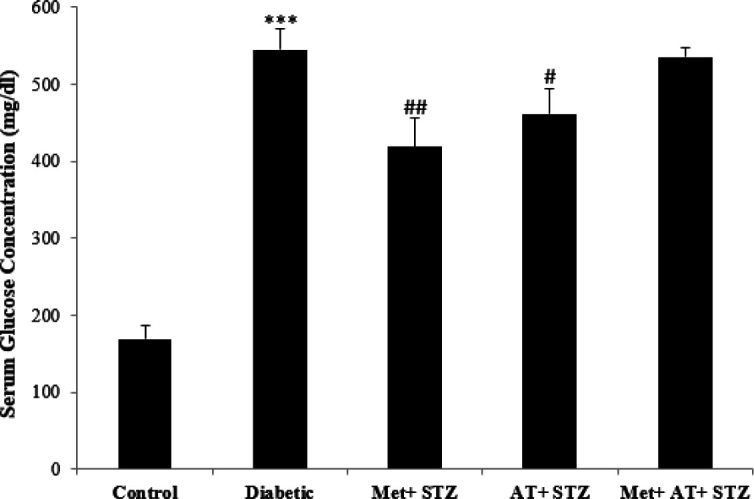
Serum glucose concentration in all experimental groups. Values are the Mean±SEM. The data was analyzed using one-way ANOVA and *post hoc* Tukey

Serum glucose concentration in diabetic group significantly increased compared with control group (p<0.001). However, treatment of diabetic rats with metformin and AT extract separately, caused a significant decrease in blood glucose compared to diabetic rats (p<0.01 and p<0.001, respectively) (Figure 1). 

***p<0.001 compared to the control group

#p<0.05 and ##p<0.01 compared to the diabetic group

Met: metformin; STZ: streptozotocin; AT: *Artemisia turanica.*

**Figure 2:**
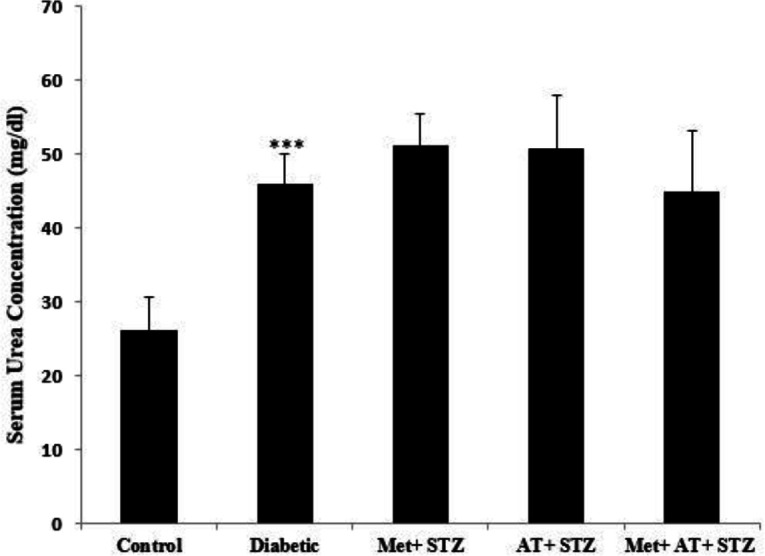
Serum urea concentration in all experimental groups. Values are the Mean±SEM. The data was analyzed using one-way ANOVA and *post hoc* Tukey

Diabetes was associated with a significant increase in serum urea concentration when compared to the control animals (p<0.001). However, administration of metformin and AT extract alone and their combination could not significantly reduce the level of serum urea compared with the diabetic group (Figure 2). 

***p<0.001 compared to the control group

Met: metformin; STZ: streptozotocin; AT: *Artemisia turanica.*

**Figure 3:**
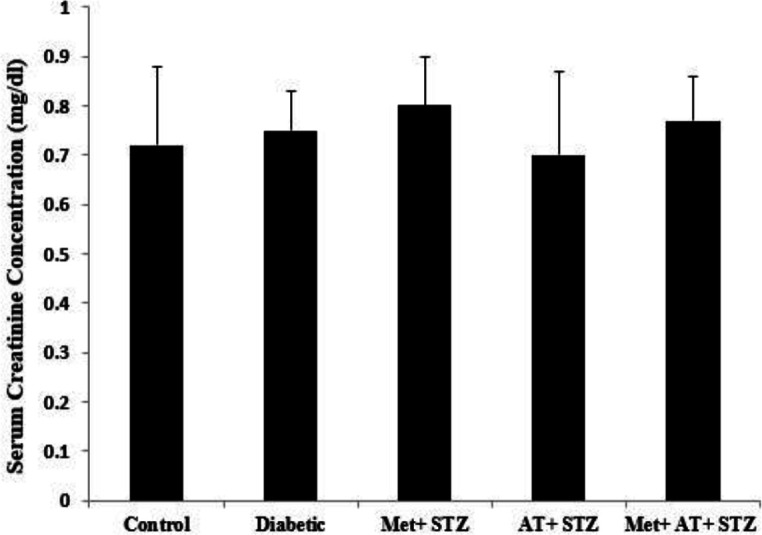
Serum creatinine concentration in all experimental groups. Values are the Mean±SEM. The data was analyzed using one-way ANOVA and *post hoc* Tukey

Serum creatinine concentration showed no significant alteration among different groups of the study (Figure 3). Alterations in serum lipid profile are summarized in Table 1. Diabetes caused significant increases in serum levels of TG when compared to the control group (p<0.01). However, serum TC and TG in metformin and AT extract-treated rats showed a significant decrease when compared to the diabetic group (p<0.05 and p<0.01). In all metformin and AT extract-treated groups, LDL-C significantly decreased when compared to the diabetic group (p<0.05 and p<0.001). Also, the level of HDL-C in Met+STZ and Met+AT+STZ groups was significantly higher than that of the diabetic group (p<0.05 for both). 

Met: metformin; STZ: streptozotocin; AT: *Artemisia turanica.*

**The** **effect of AT extract and metformin on kidney tissue oxidative stress markers**

**Figure 4.**
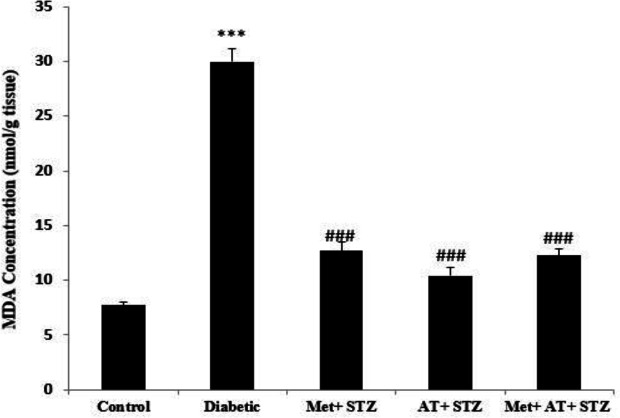
Renal MDA concentration in all experimental groups. Values are the Mean±SEM. The data was analyzed using one-way ANO VA and *post hoc* Tukey

**Figure 5.**
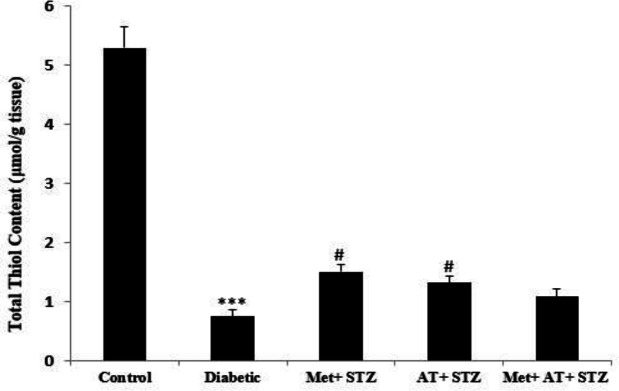
Renal total thiol content in all experimental groups. Values are the Mean±SEM. The data was analyzed using one-way ANOVA and *post hoc* Tukey

Figure 4 shows the renal MDA concentration in different groups. In diabetic rats, MDA concentration was significantly higher than the control animals (p<0.001; Figure 4). However, in all metformin and AT extract-treated rats, renal MDA levels significantly decreased when compared to the diabetic group (p<0.001 for all; Figure 4). Diabetes was also associated with a significant decrease in total thiol content when compared to the control animals (p<0.001; Figure 5). However, separate treatment of diabetic rats with AT extract and metformin significantly increased total thiol concentration compared with the diabetic group (p<0.05 for both; Figure 5).

***p<0.001 compared to the control group

^###^p<0.001 compared to the diabetic group

Met: metformin; STZ: streptozotocin; AT: *Artemisia turanica.*

***p<0.001 compared to the control group

#p<0.05 compared to the diabetic group

Met: metformin; STZ: streptozotocin; AT: *Artemisia turanica.*

**Figure 6:**
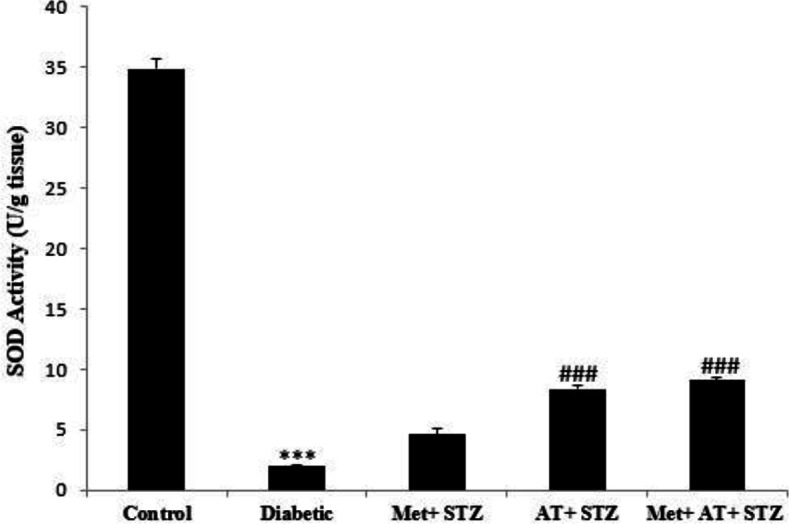
Renal superoxide dismutase (SOD) activity in all experimental groups. Values are the Mean±SEM. The data was analyzed using one-way ANOVA and *post hoc* Tukey

**Figure 7:**
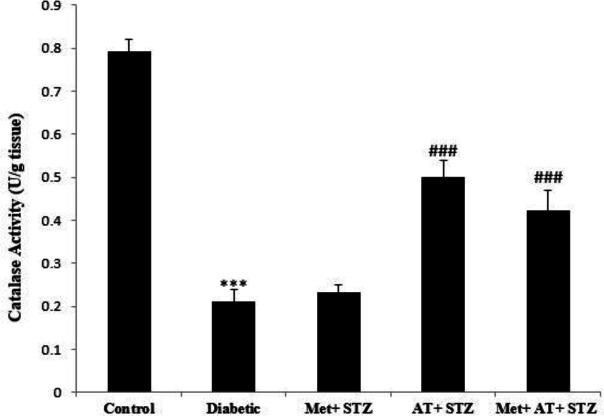
Renal catalase activity in all experimental groups. Values are the Mean±SEM. The data was analyzed using one-way ANOVA and *post hoc* Tukey

Figures 6 and 7 demonstrate the activities of SOD and catalase enzymes in kidney tissue in all experimental groups. The results showed that SOD and catalase activities significantly decreased in the diabetic group compared to the control group (p<0.001). Interestingly, unlike metformin, AT extract could significantly increase the activities of these two antioxidant enzyme in kidney tissues of STZ-induced diabetic rats (p<0.001 for all) (Figures 6 and 7). 

***p<0.001 compared to the control group

^###^p<0.001 compared to the diabetic group

Met: metformin; STZ: streptozotocin; AT: *Artemisia turanica.*

***p<0.001 compared to the control group

^###^p<0.001 compared to the diabetic group

Met: metformin; STZ: streptozotocin; AT: *Artemisia turanica.*

**Table 1:** The effect of *Artemisia turanica* on serum lipid profile in all experimental groups (n=8).

	**Triglyceride** ** (mg/dl)**	**Total Cholesterol (mg/dl)**	**LDL** **(mg/dl)**	**HDL** **(mg/dl)**
Control Diabetic Met+STZ AT+STZ Met+AT+STZ	81.66±8.04 116.2±10.38** 66±1.22++ 59.66±8.16++ 86.4±9.16	77.4±8.87 85±5.2 67.66±8.35*+ 65.5±7.18*+ 73.66±4.71	26.83±3.12 30.2±3.07 23.5±0.86*+ 15.75±1.49+++ 19.2±0.65*++	21±1.26 24.5±2.04 30.83±4.27**+ 26.16±1.7 34.66±2.87**+

Values are the Mean±standard error of the mean. Values are the Mean±SEM. The data was analyzed using one-way ANOVA and *post hoc* Tukey. A significant difference was considered at p<0.05. *p<05, **p<01 compared to the control group. +p<05, ++p<01, and +++p<001 compared to the diabetic group

## Discussion

Diabetic nephropathy is a chronic complication of diabetes mellitus. Although the precise mechanisms of diabetic nephropathy are not well understood, various studies emphasized the important role of microvascular disturbances that are caused due to the hyperglycemia-induced oxidative stress. In fact, oxidative stress is regarded as the major factor linking hyperglycemia with vascular complications (Brownlee, 2001 ▶; Papaharalambus and Griendling, 2007 ▶). The present study showed diabetes-induced oxidative damage to the kidneys as evidenced by significant changes in MDA, thiol and renal activities of SOD and catalase. In the current study, we found that treatment of diabetic rats with AT extract could reduce MDA levels and increase total thiols and antioxidant enzymes activities in kidney homogenate. In a recent study, we showed the antioxidant effect of AT extract on liver oxidative damage in diabetic rats (Bagheri Yazdi et al., 2019 ▶). Also, some previous studies described the antioxidant effect of different species of *Artemisia* (Hallal et al., 2016 ▶; Selmi et al., 2016 ▶). Therefore, according to the results of the present study, the beneficial effect of AT extract on diabetes-induced renal oxidative stress might be due to its antioxidant effects mainly by terpenoid compounds (Bagheri Yazdi et al., 2019 ▶). Interestingly, in our study, the effect of AT extract, alone and in combination with metformin, was more prominent than that of metformin on increasing the activity of SOD and catalase. However, similar to serum glucose, the effect of separate administration of AT extract on catalase activity was more prominent than the effect of its combination with metformin. Although the mechanism of these findings is unclear, AT extract probably has no notable synergistic effect with metformin, as they reduce the beneficial effects of each other in these cases. Our results suggest that renal function, in terms of serum urea and creatinine concentrations, showed no significant change in diabetic and nondiabetic rats; this finding is supported by other studies (Li et al., 2014 ▶; Chen et al., 2017 ▶; Zhang et al., 2017 ▶). This nonsignificant effect of AT on serum urea and creatinine may be due to duration of the experiment which was insufficient for induction of kidney dysfunction.

It has long been demonstrated that hyperlipidemia causes renal injury and aggravates the progression of diabetic nephropathy (Kachhawa et al., 2017 ▶). Abnormal serum lipid level increases extracellular matrix deposition and cytokine production by mesangial cells (Kachhawa et al., 2016 ▶). Hypercholesterolemia and deposition of LDL cause glomerulosclerosis through macrophage infiltration and foam cell formation (Kachhawa et al., 2016 ▶). In our study, plasma TG level significantly increased in STZ diabetic rats, but plasma HDL-C, LDL-C and total cholesterol showed no significant change compared to the normal rats. However, separate administration of metformin and AT extract prominently reduced serum TG, LDL-C and cholesterol in diabetic rats. Co-administration of metformin and AT extract caused a significant increase in serum HDL-C and a significant decrease in serum LDL-C. However, combination therapy with metformin and AT extract reduced serum cholesterol and TG by 14 and 26%, respectively. Previous studies suggested that some species of *Artemisia* including *Artemisia vulgaris *and* Artemisia sieberi* have hypolipidemic effect (El-Tantawy, 2015 ▶; Mansi et al., 2007 ▶). Phytochemical analysis of *Artemisia turanica* extract revealed the presence of tannins, flavonoids, terpenoids and steroids. Therefore, hypolipidemic effect of AT extract might possibly be due to the presence of these hypolipidemic compounds. 

In conclusion, the findings of the present study revealed that AT extract possesses antioxidant properties, as well as hypolipidemic effects in diabetic rats. Further investigations are required to elucidate mechanisms of the beneficial actions of AT extract on diabetes.

## References

[B1] Aebi H (1984). Catalase in vitro. Methods in enzymology.

[B2] Bagheri Yazdi H, Hojati V, Shiravi A, Hosseinian S, Vaezi G, Hadjzadeh MR (2019). Liver Dysfunction and Oxidative Stress in Streptozotocin-Induced Diabetic Rats: Protective Role of Artemisia Turanica. J Pharmacopuc.

[B3] Behravan J, Ramezani M, Hassanzadeh M, Eliaspour N, Sabeti Z (2006). Cytotoxic and Antimycotic Activities of essential oil of Artemisia turanica Krasch from Iran. J Essent Oil Bear Plants.

[B4] Biesenbach G (1989). Disorders of lipid metabolism in diabetes mellitus. Wien Med Wochenschr Suppl.

[B5] Brownlee M (2001). Biochemistry and molecular cell biology of diabetic complications. Nature.

[B6] Chawla T, Sharma D, Singh A (2010). Role of the renin angiotensin system in diabetic nephropathy. World J Diabetes.

[B7] Chen X, Wu R, Kong Y, Yang Y, Gao Y, Sun D, Qizhen L, Dongjun D, Zeyuan L, Niansong W, Sheng G, Feng W (2017). Tanshinone II Aattenuates renal damage in STZ-induced diabetic rats via inhibiting oxidative stress and inflammation. Oncotarget.

[B8] Cheng H, Harris R (2014). Renal endothelial dysfunction in diabetic nephropathy. Cardiovasc Hematol Disord Drug Targets.

[B9] Dashtban M, Sarir H, Omidi A (2016). The effect of Prosopis farcta beans extract on blood biochemical parameters in streptozotocin-induced diabetic male rats. Adv Biomed Res.

[B10] El-Tantawy WH (2015). Biochemical effects, hypolipidemic and anti-inflammatory activities of Artemisia vulgaris extract in hypercholesterolemic rats. J Clin Biochem Nutr.

[B11] Forbes JM, Coughlan MT, Cooper ME (2008). Oxidative stress as a major culprit in kidney disease in diabetes. Diabetes.

[B12] Hallal N, Kharoubi O, Benyettou I, Tair K, Ozaslan M, Aoues A (2016). In vivo amelioration of oxidative stress by Artemisia absinthium L administration on mercuric chloride toxicity in brain regions. J Biol Sci.

[B13] Hosseinian S, Ebrahimzadeh Bideskan A, Shafei MN, Sadeghnia HR, Soukhtanloo M, Shahraki S, Samadi Noshahr Z, Khajavi Rad A (2018). Nigella sativa extract is a potent therapeutic agent for renal inflammation, apoptosis, and oxidative stress in a rat model of unilateral ureteral obstruction. Phytother Res.

[B14] Hosseinian S, Khajavi Rad A, Ebrahimzadeh Bideskan A, Soukhtanloo M, Sadeghnia HR, Shafei MN, Motejadded F, Mohebbati R, Shahraki S, Beheshti F (2017). Thymoquinone ameliorates renal damage in unilateral ureteral obstruction in rats. Pharmacol Rep.

[B15] Hosseinzadeh L, Malekshahi A, Ahmadi F, Emami A, Hajialyani M, Mojarrab M (The Protective Effect of Different Extracts of Three Artemisia Species against H2O2-Induced Oxidative Stress and Apoptosis in PC12 Neuronal Cells). 2018. Pharmacogn Res.

[B16] Kachhawa K, Agrawal D, Rath B, Kumar S (2017). Association of lipid abnormalities and oxidative stress with diabetic nephropathy. J Integr Nephrol Androl.

[B17] Kachhawa K, Varma M, Kachhawa P, Sahu A, Shaikh M, Kumar S (2016). Study of dyslipidemia and cystatin C levels as a predictive marker of chronic kidney disease in type 2 diabetes mellitus patients at a teaching hospital in Central India. J Integr Nephrol Androl.

[B18] Kashihara N, Haruna Y, K Kondeti V, S Kanwar Y (2010). Oxidative stress in diabetic nephropathy. Curr Med Chem.

[B19] Khayyat MH, Karimi H (2005). Composition of the volatile oils of three different species of Artemisia. Iran J Pharm Sci.

[B20] Li W, Wang G, Lu X, Jiang Y, Xu L, Zhao X (2014). Lycopene ameliorates renal function in rats with streptozotocin-induced diabetes. Int J Clin Experimental Pathol.

[B21] Madesh M, Balasubramanian K (1998). Microtiter plate assay for superoxide dismutase using MTT reduction by superoxide. Indian J Biochem Biophys.

[B22] Mansi K, Amneh M, Nasr H (2007). The hypolipidemic effects of Artemisia sieberi (A herba-alba) in alloxan induced diabetic rats. Int J Pharmacol.

[B23] Mozaffarian V (1998). A Dictionary of Iranian Plant Names.

[B24] Papaharalambus CA, Griendling KK (2007). Basic mechanisms of oxidative stress and reactive oxygen species in cardiovascular injury. Trends Cardiovasc Med.

[B25] Park MH, Han JS (2012). Hypoglycemic Effect of Padina arborescens Extract in Streptozotocin-induced Diabetic Mice. Prev Nutr Food Sci.

[B26] Pourghasem M, Shafi H, Babazadeh Z (2015). Histological changes of kidney in diabetic nephropathy. Caspian J Intern Med.

[B27] Reutens AT, Atkins RC (2011). Epidemiology of diabetic nephropathy. Diabetes and the Kidney.

[B28] Selmi S, Rtibi K, Grami D, Hajri A, Hosni K, Marzouki L, Sebai, H (2016). Antioxidant properties of Artemisia herba-alba and Eucalyptus camaldulensis essentials oils on malathion-induced reproductive damage in rat. RSC Adv.

[B29] Taherkhani M, Rustaiyan A, Nahrevanian H, Naeimi S, Taherkhani T (2013). Comparison of antimalarial activity of Artemisia turanica extract with current drugs in vivo. J Vector Borne Dis.

[B30] Yun C, Jung Y, Chun W, Yang B, Ryu J, Lim C, Kim JH, Kim H, Cho SI (2016). Anti-Inflammatory Effects of Artemisia Leaf Extract in Mice with Contact Dermatitis In Vitro and In Vivo. Mediators Inflamm.

[B31] Zhang S, Xu H, Yu X, Wu Y, Sui D (2017). Metformin ameliorates diabetic nephropathy in a rat model of low-dose streptozotocin-induced diabetes. Exp Ther Med.

